# Pseudouridine Detection and Quantification using Bisulfite Incorporation Hindered Ligation

**DOI:** 10.1021/acschembio.4c00387

**Published:** 2024-07-16

**Authors:** Yutao Zhao, Xinyuan Ma, Chang Ye, Wenlong Li, Kinga Pajdzik, Qing Dai, Hui-Lung Sun, Chuan He

**Affiliations:** a Department of Chemistry, The University of Chicago, Chicago, Illinois 60637, USA; b Howard Hughes Medical Institute, The University of Chicago, Chicago, Illinois 60637, USA

**Keywords:** epitranscriptomics, pseudouridine, RNA modification, next-generation sequencing

## Abstract

Pseudouridine (Ψ) is a widespread RNA modification found in various RNA species, including rRNA, tRNA, snRNA, mRNA, and long noncoding RNA (lncRNA). Understanding the function of Ψ in these RNA types requires a robust method for detection and quantification of the Ψ level at single-nucleotide resolution. A previously used method utilizes Ψ labeling by N-cyclohexyl-N’-β-(4-methylmorpholinium)ethylcarbodiimide (CMC). The quantification of Ψ is based on the stop ratio after reverse transcription. However, the use of CMC followed by strong alkaline treatment causes severe RNA degradation, often requiring a large amount of RNA. The removal of CMC and recovery of RNA by ethanol precipitation are also time-consuming. Here, we introduce a Bisulfite Incorporation Hindered ligation-based method (BIHIND) which can detect and quantify Ψ sites on rRNA, mRNA and non-coding RNA. BIHIND can be coupled with quantitative PCR (BIHIND-qPCR) for quantitative detection of Ψ fraction at individual modification site, as well as with next-generation sequencing (BIHIND-seq) for high-throughput sequencing of Ψ without requiring reverse transcription. We have validated the robustness of BIHIND with the elucidation of Ψ dynamics following pseudouridine synthase depletion.

## Graphical Abstract

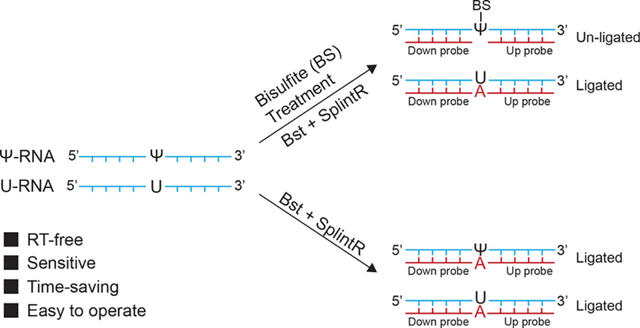

## Introduction

Pseudouridine (Ψ), often referred as the ‘fifth nucleotide’ of RNA, is one of most abundant post-transcriptional RNA modifications within rRNA, tRNA, and snRNA (small nuclear RNA)^1–2^. Ψ is characterized by its C–C glycosidic isomerization of uridine (U), which endows Ψ with an extra N–H hydrogen bond donor on the non-Watson-Crick edge and contributes to RNA folding^3^. Ψ is involved in many important cellular metabolic processes. Depletion of Ψ on rRNA decreases translation initiation and translational fidelity^4^. Recently, Ψ incorporated by PUS7 (pseudouridine synthase 7) on tRNA is reported to regulate a network of tRNA-derived small fragments (tRFs), which affect translation initiation in human stem cells^5^. These Ψ-derived tRNA fragments also play roles in codon-specific translational control in Glioblastoma^6^. Dysregulation of Ψ may also contribute to human diseases. Decreased expression of a snoRNA, SNORA24, which guides rRNA pseudouridylation, leads to reduced Ψ on rRNA and contributed to liver cancer^7^. This base modification also plays critical roles in mammalian development and many other biological processes. Accurate detection and quantification of Ψ on RNA are therefore important to functional investigations.

In the last decade, significant progresses have been made in the development of methods for detecting Ψ in RNA. Detection and quantification of Ψ have relied heavily on Ψ labeling by N-cyclohexyl-N’-β-(4-methylmorpholinium)ethylcarbodiimide (CMC) to produce CMC-modified Ψ^8–12^. While this approach enables detection of Ψ at the base resolution, quantification of Ψ level presents challenges. The process of CMC treatment, followed by alkaline treatment, also induces substantial RNA degradation. Besides, removal of CMC and recovery of RNA are time-consuming. Consequently, a significant amount of RNA is required. Recent efforts have been made in optimizing CMC-based Ψ detection, employing lower reaction temperatures to improve RNA recovery^11^. However, such optimizations often compromise time efficiency, and accurate quantification is still an issue.

**Scheme 1.**
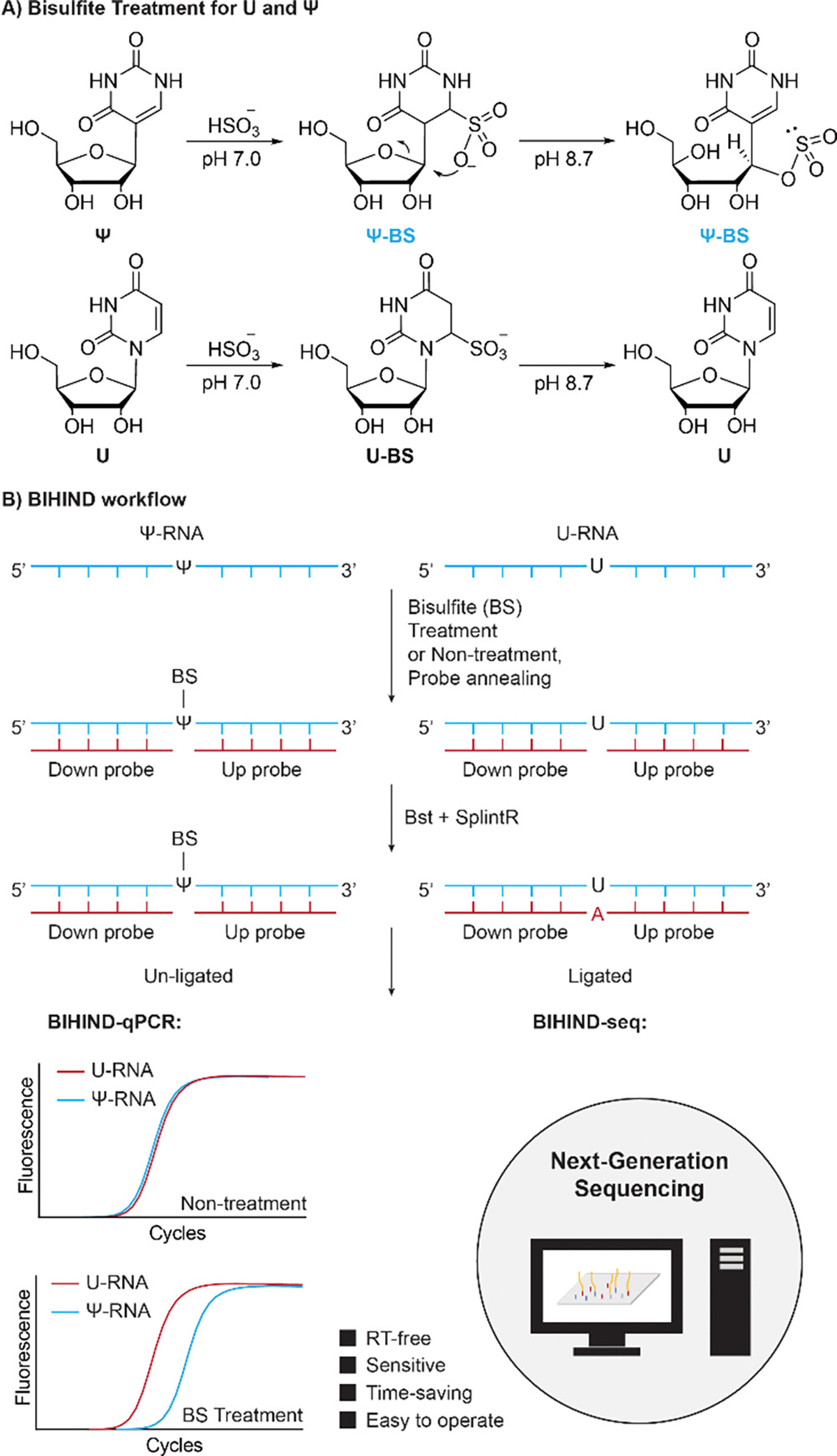
Illustrations of bisulfite (BS) reactivity towards Ψ and U, and BIHIND workflow. A) Ψ-containing RNA is labeled by BS. B) Ψ-BS is expected to hinder elongation of Bst polymerase or ligation of SplintR ligase. The successful occurrence of both elongation and ligation events enables the detection of the final ligated product through quantitative PCR (BIHIND-qPCR) or next-generation sequencing (BIHIND-seq).

The SELECT method has emerged as a powerful tool for detecting RNA modifications like N6-methyladenosine (m^6^A), N1-methyladenosine (m^1^A) and 2’-O-methyladenosine (A_m_)^13^. In this method, two DNA oligos, the “up probe” and “down probe”, are annealed to RNA templates upstream and downstream of a putative m^6^A site, respectively, leaving a 1-nt gap opposite the target site, which enables the detection of the m^6^A modification. However, the structural similarity between U and Ψ poses challenges in distinguishing between these two nucleosides. The recent developments of the BID-seq and PRAISE method by Dai and Zhang *et al.* offer a solution of distinguishing Ψ and U through bisulfite treatment at neutral pH^14–15^. This approach utilizes sodium bisulfite (BS) to form Ψ-BS without affecting U, allowing for discrimination between these two nucleosides. In principle, sodium bisulfite (BS) at neutral pH can undergo Michael addition with U and Ψ to form U-BS and Ψ-BS without reacting with cytosine. The following slightly alkaline treatment reverses U-BS to U, while Ψ-BS remains stable (Scheme 1, Figure S1a–S1c). Therefore, we could combine BS treatment and SELECT method to achieve Ψ detection and quantification.

Here, based on the previously reported SELECT method, we present a Bisulfite Incorporation Hindered Ligation-based method (BIHIND) for detection and quantification of Ψ (scheme 1). It takes advantages of two features of Ψ-BS: 1) BS incorporation hinders single-base elongation by DNA polymerase; 2) BS incorporation hinders the nick ligation of ligases (Scheme 1). This approach, coupled with quantitative PCR (BIHIND-qPCR) or next-generation sequencing (BIHIND-seq), offers a robust platform for detecting and quantifying Ψ sites in low-input samples across various RNA species. Furthermore, we validate the utility of BIHIND by assessing the altered fraction of Ψ sites following the knockdown of pseudouridine synthases, PUS7, TRUB1 and DKC1, demonstrating its application in elucidating Ψ dynamics.

## Results

**Figure 1.**
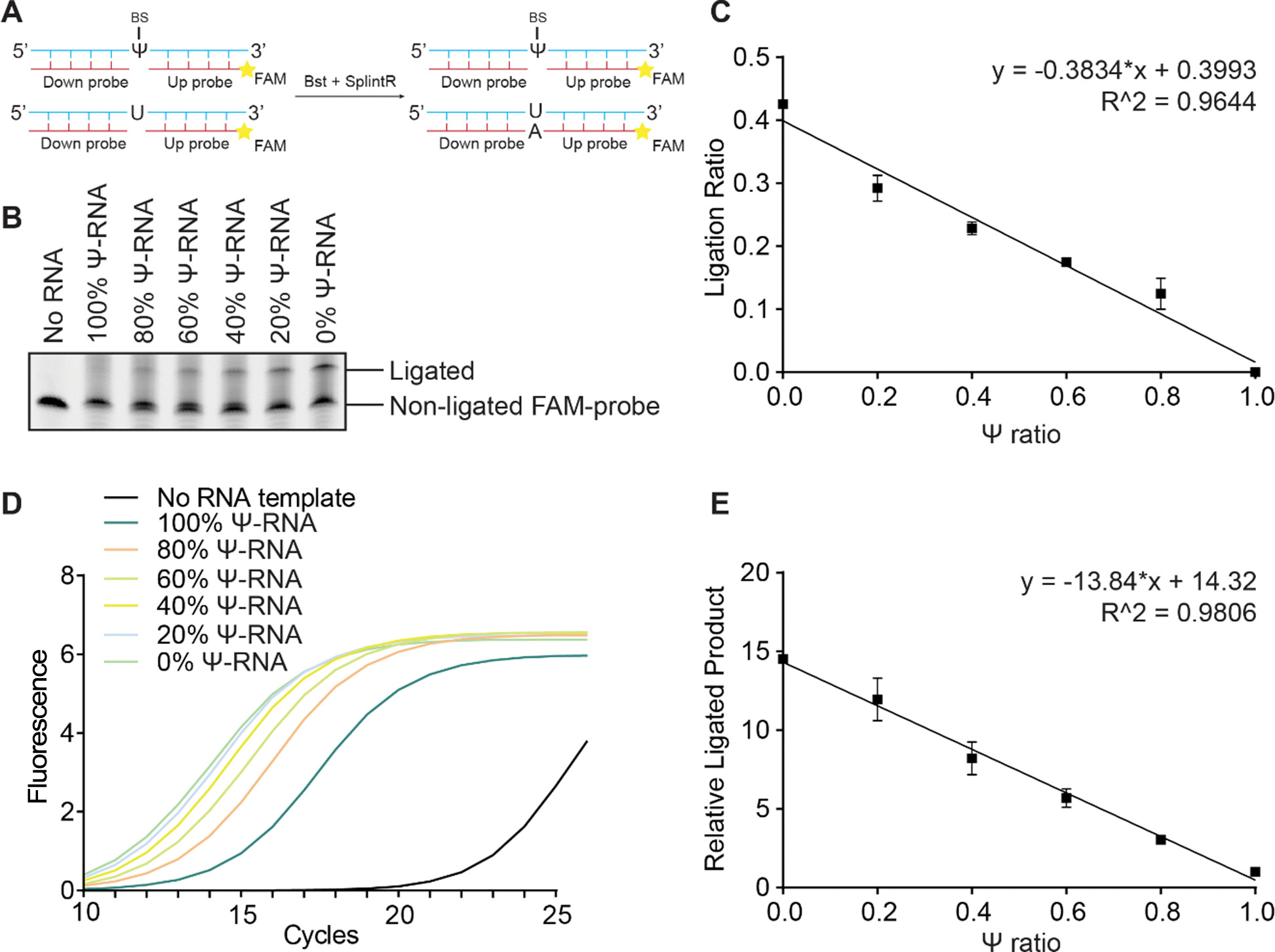
Validation of the BIHIND method for Ψ detection using 41-mer RNA oligo. A) Proof-of-concept design of BIHIND. 50 ng of the oligo was treated with BS at 70 °C for 3 hours, “up- and downprobes” of 1 μM were annealed, and elongation and ligation were performed according to an improved SELECT protocol. B) PAGE analysis showing the ligation efficiency of different RNA oligo template with various Ψ fractions. C) The linear relationship between ligation ratio and Ψ fractions. D) Real-time fluorescence amplification curves showing BIHIND results for detecting various Ψ fractions. E) The linear relationship between ligated products and corresponding Ψ fractions. Error bars, for 2 biological replicates × 2 technical replicates.

We initiated our investigation by assessing the efficiency of the SELECT protocol using 41-mer synthetic RNA oligos containing either Ψ or U in the middle. Without bisulfite (BS) treatment, the original SELECT method failed to distinguish between Ψ and U probes via qPCR (Figure S2). We hypothesized that the incorporation of BS at Ψ site could increase its bulkiness, thus impeding the elongation and ligation processes. To validate this hypothesis, we designed a FAM (Fluorescein amidites)-labeled “up probe” and a 5’-phosphorylated “down probe”, which could anneal to the 41-mer RNA oligo. After incubating the 41-mer oligo with various Ψ fractions in BS reagent at 70 °C for 3 hours^14^, and annealing the designed “up probe” and “down probe” (Figure 1A), we followed an improved SELECT protocol^16^ by Wang *et al* and treated the RNA-DNA hybrid with Bst 2.0 polymerase and subsequently with SplintR ligase. The crude product underwent polyacrylamide gel electrophoresis (PAGE) analysis. The results revealed that 0% Ψ-RNA yielded a 40% ligated product, whereas no product was observed in the case of 100% Ψ-RNA (Figure 1B). This indicates that BS incorporation can completely inhibit the elongation and ligation process when using Bst and SplintR. The ligation efficiency demonstrated a perfect linear correlation with the Ψ fraction (Figure 1C). Furthermore, we validated our findings through BIHIND-qPCR, where the ligated product also displayed a strong linear relationship, affirming the efficacy of our approach in distinguishing between Ψ and U (Figure 1D and 1E).

**Figure 2.**
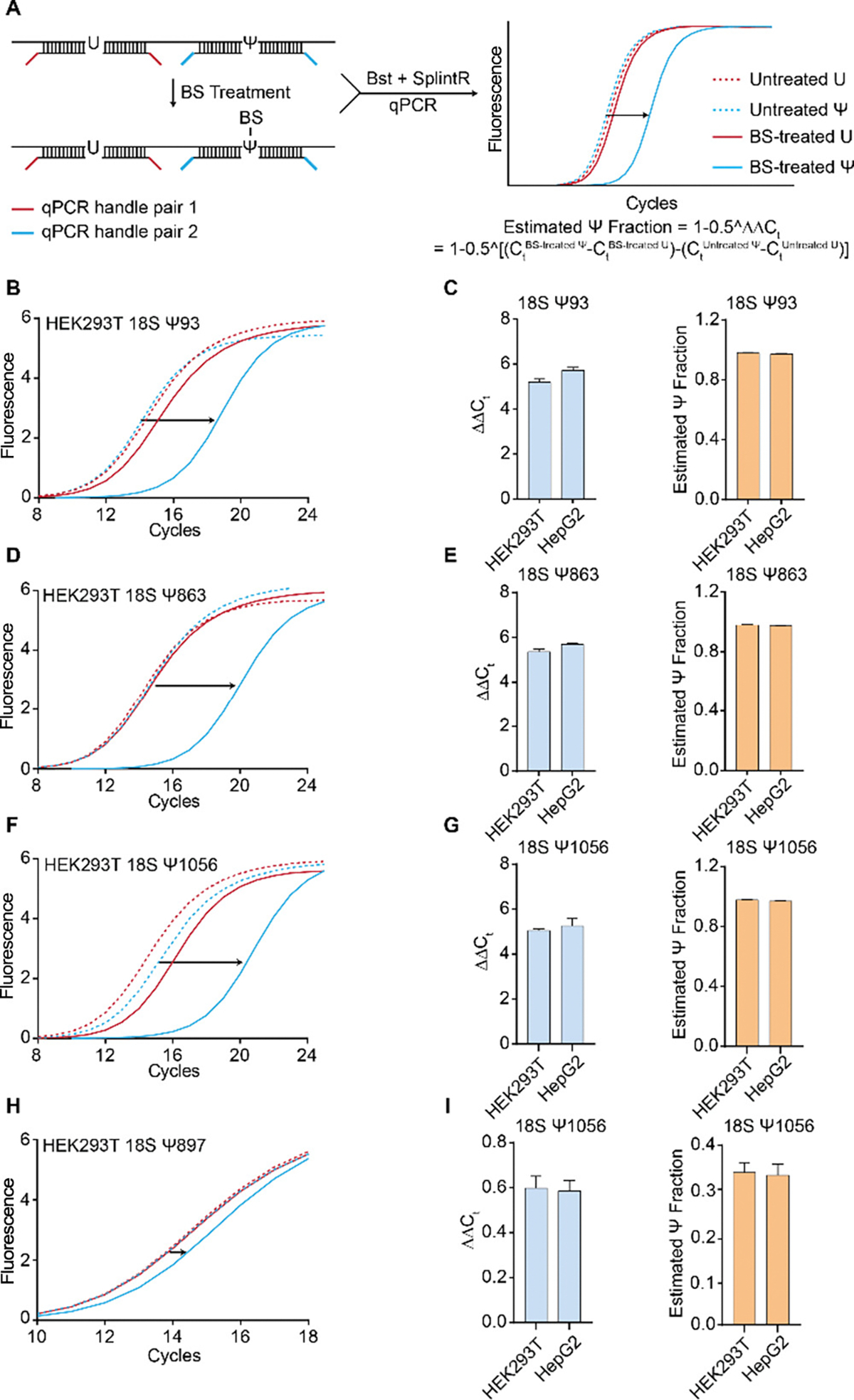
BIHIND-qPCR used for determining Ψ fraction at ribosomal RNA 18S Ψ93, Ψ863, Ψ1056 and Ψ897, using 100 ng of total RNA. A) BIHIND-qPCR workflow and estimation of Ψ fraction. B) Real-time fluorescence amplification curves of BIHIND-qPCR for detecting Ψ in HEK-293T 18S rRNA Ψ93. C) Calculations of ΔΔC_t_ and Ψ fractions of 18S rRNA Ψ93 in HEK-293T and HepG2 cells. D) Real-time fluorescence amplification curves of BIHIND-qPCR for detecting Ψ in HEK-293T 18S rRNA Ψ863. E) Calculations of ΔΔC_t_ and Ψ fractions of 18S rRNA Ψ863 in HEK-293T and HepG2 cells. F) Real-time fluorescence amplification curves of BIHIND-qPCR for detecting Ψ in HEK-293T 18S rRNA Ψ1056. G) Calculations of ΔΔC_t_ and Ψ fractions of 18S rRNA Ψ1056 in HEK-293T and HepG2 cells. Error bars for 2 biological replicates × 3 technical replicates. H) Real-time fluorescence amplification curves of BIHIND-qPCR for detecting Ψ in HEK-293T 18S rRNA Ψ897. I) Calculations of ΔΔC_t_ and Ψ fractions of 18S rRNA Ψ897 in HEK-293T and HepG2 cells. Error bars for 2 biological replicates × 3 technical replicates.

Following the successful validation of BIHIND design using 41-mer probes, we proceeded to verify the BIHIND-qPCR approach on biological samples. Given the abundant pseudouridylation of rRNA in mammalian cells, we selected three known Ψ sites (18S Ψ93, Ψ863, Ψ1056) on HEK-293T 18S rRNA with close to 100% fraction. Recognizing that the initial RNA input and RNA recovery variance after BS treatment can influence qPCR amplification, we designed probes targeting both U and Ψ sites for normalization purposes (Figure 2A). We define ΔΔC_t_ as C_t_ normalized change for Ψ after treatment and estimate Ψ fraction for that site as 1−0.5^ΔΔC_t_. With or without BS treatment, the qPCR amplification curve exhibited minor change for the U site, while for the Ψ site, it shifted to a higher C_t_ value (Figure 2B, 2D, 2F). This indicates that BS incorporation indeed hinders the elongation and ligation processes of Bst and SplintR in biological samples. After calculating ΔΔC_t_, we estimated ≥97% Ψ modification level for all these three sites (Figure 2C, 2E, 2G), which corresponds well with previously reported level^14^. A lowly Ψ-modified site in 18S rRNA Ψ897, which was previously reported to have a 26% pseudouridine fraction, was quantified as 35% using BIHIND-qPCR (Figure 2H, 2I). Additionally, another set of BIHIND-qPCR data from the second cell line, HepG2, indicated conservation of these three Ψ sites on rRNA (Figure 2C, 2E, 2G).

**Figure 3.**
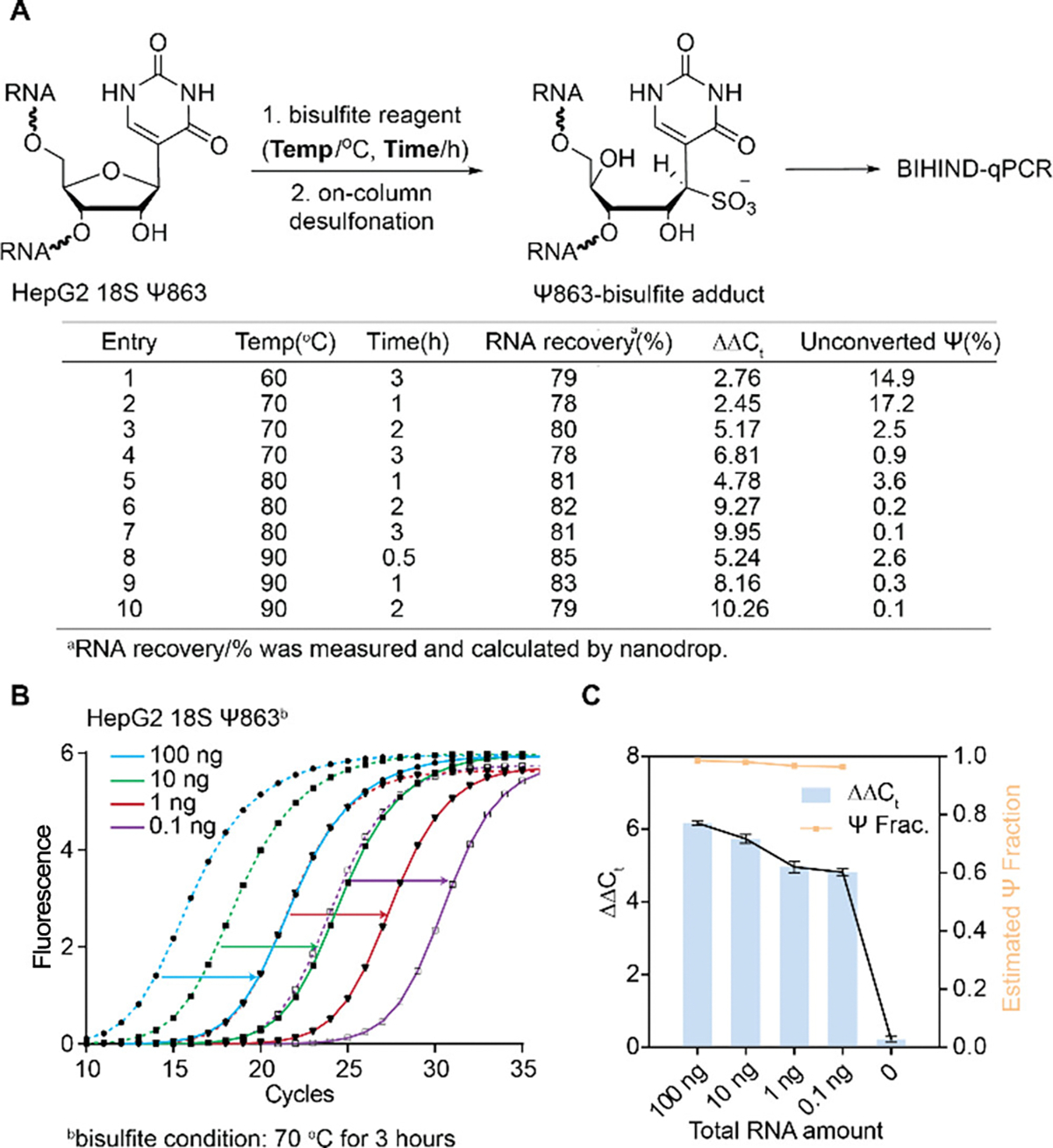
Evaluation of BS reaction condition towards Ψ and BIHIND-qPCR performance with a series of RNA dilutions. A) Evaluation of different temperature and time of BS reaction. 1 μg total RNA was used in this experiment. RNA recovery was measured and calculated by nanodrop. Unconverted Ψ/% was calculated as 100x(1−0.5^ΔΔC_t_). B) Real-time fluorescence amplification curves of BIHIND-qPCR on a series of RNA dilutions ranging from 100 ng to 0.1 ng. C) ΔΔC_t_ and estimated Ψ fraction alternations as the dilution fold increases. Error bars, mean±s.d. for 2 biological replicates × 3 technical replicates.

We next studied18S rRNA Ψ863 from HepG2 cells using BIHIND-qPCR. We explored a range of temperatures, from 60°C to 90°C, across various incubation time to derive ΔΔC_t_ values and quantify unconverted Ψ under each BS condition. Our findings revealed that reducing the temperature to 60°C for 3 hours resulted in a modest Ψ conversion rate of 85.1% (Figure 3A, Entry 1), probably due to the persistence of unopened secondary structures in the 18S RNA at this temperature, as evidenced by RNA electrophoresis (Figure S3). By contrast, elevating the reaction temperature and extending the reaction time yielded optimal Ψ conversion rates (Figure 3A, Entries 4, 6, 7, 9, 10), without introducing any change to uridine or cytosine (Figure S1b, S1c). Although these conditions led to varying degrees of RNA degradation (Figure S3), the overall RNA recovery remained unaffected (Figure 3A, Entries 4, 6, 7, 9, 10), offering an opportunity to save reaction time by increasing temperature without compromising detection accuracy. Furthermore, increased RNA dilution fold for BIHIND-qPCR didn’t cause significant alterations in ΔΔC_t_ values and estimated Ψ fractions (Figure 3B, 3C), underscoring the great potential of our method for accurately detecting Ψ in low-input RNA samples.

**Figure 4.**
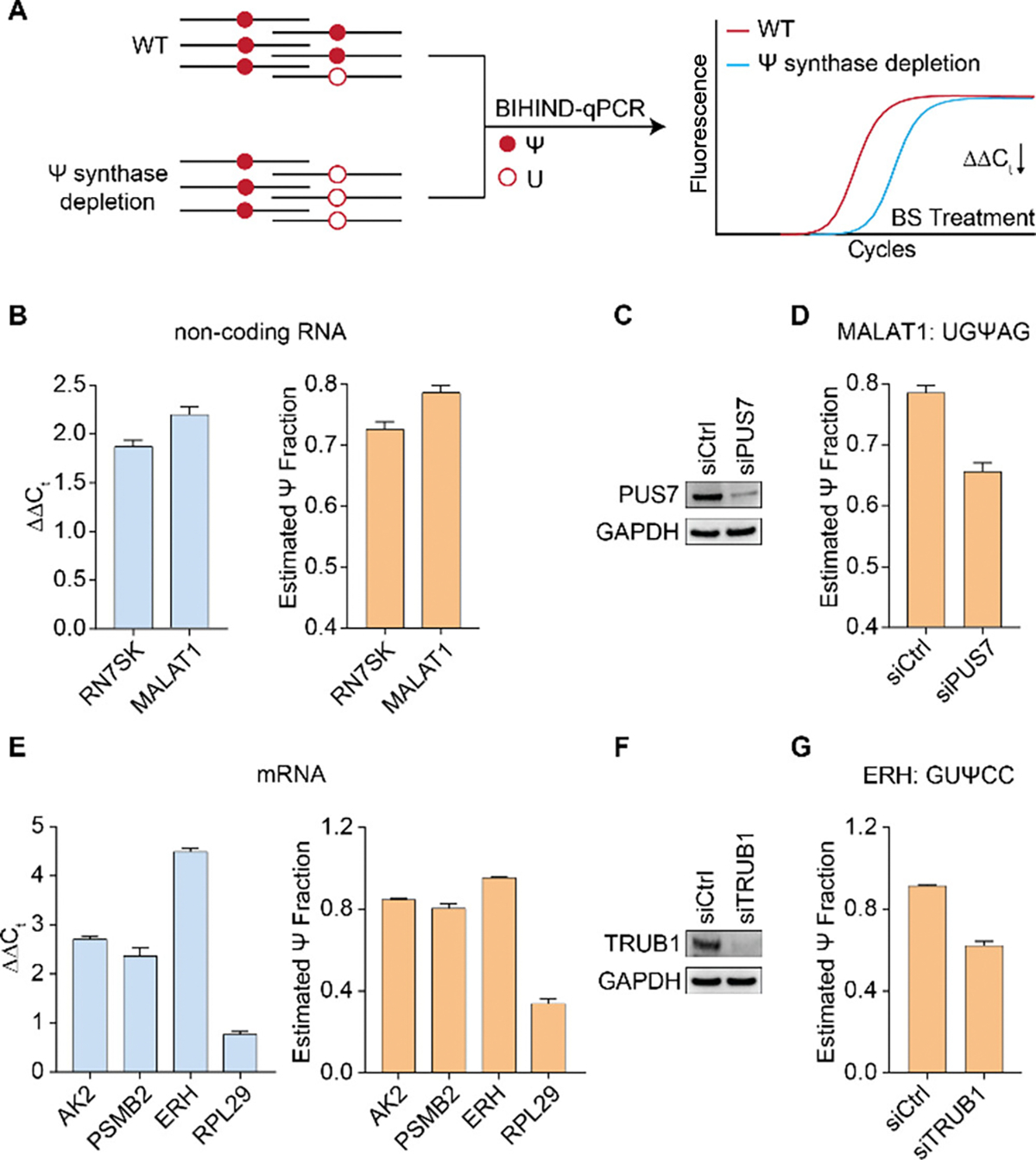
BIHIND-qPCR used for Ψ site detection on non-coding RNA and low-abundance mRNA. A) Workflow for identification of Ψ synthase responsible for installing specific Ψ site. B) Quantification of Ψ fractions on *RN7SK* Ψ250 and *MALAT1* Ψ5614 by BIHIND-qPCR, using 300 ng Hela total RNA. C) Western blot showing reduced PUS7 expression level with siPUS7 treatment. D) Bar plot showing reduced Ψ fraction for *MALAT1* Ψ5614 after PUS7 depletion. E) Quantification of Ψ fractions on *AK2* Ψ522, *PSMB2* Ψ634, *ERH* Ψ649 and RPL29 Ψ380 by BIHIND-qPCR, using 50 ng Hela mRNA. F) Western blot showing reduced TRUB1 expression level with siTRUB1 treatment. G) Bar plot showing reduced Ψ fraction for *ERH* Ψ649 after *TRUB1* depletion. Error bars, mean±s.d. for 2 biological replicates × 3 technical replicates.

With robust Ψ detection method in hand, we proceeded to assess the efficacy of BIHIND in validating and quantifying known Ψ sites on both non-coding RNA and mRNA. RN7SK, a prevalent small nuclear RNA in mammalian cells regulates transcription by modulating the positive transcription elongation factor P-TEFb^17–18^. *MALAT1*, a highly conserved nuclear lncRNA, exhibits abundant expression and plays diverse regulatory roles at transcriptional and post-transcriptional levels in various contexts^19^. Previous reports identified Ψ250 on *RN7SK* and Ψ5614 on *MALAT1* transcripts using Pseudo-seq, albeit lacking quantitative information^9^. Utilizing the BIHIND-qPCR method, we quantified the modification levels of *RN7SK* Ψ250 at 73% and *MALAT1* Ψ5614 at 78% (Figure 4B). Notably, the calculated *MALAT1* Ψ5614 level closely aligns with the one obtained by the CMC-based qPCR method^11^, validating the reliability of BIHIND. Moreover, BIHIND can be readily applied to study Ψ dynamics, such as assigning Ψ synthase for specific Ψ sites (Figure 4A). Considering *MALAT1*’s Ψ5614 harbors the consensus motif UGΨAG, which is a preferred motif for pseudouridine synthase 7 (PUS7). We transiently knocked down PUS7 in Hela cells using siRNA (Figure 4C), and observed a reduction in the Ψ fraction from 78% to 65% for *MALAT1* Ψ5614 using BIHIND-qPCR (Figure 4D). This suggests the involvement of PUS7 in Ψ installation at this site for the *MALAT1* transcript.

To illustrate the applicability of our method to low-abundance mRNA, we targeted four Ψ sites (*AK2* Ψ522, *PSMB2* Ψ634, *ERH* Ψ649) located on adenylate kinase 2 (*AK2*), proteasome 20S subunit beta 2 (*PSMB2*), Enhancer of Rudimentary Homolog (*ERH*) and Ribosomal Protein L29 (*RPL29*) in HeLa cells, which displayed 100%, 100%, 100%, 51% Ψ fraction, respectively, according to the reported BID-seq data^14^. Using 50 ng of Hela mRNA, we assessed the Ψ380 level of *AK2* Ψ522, *PSMB2* Ψ634, *ERH* Ψ649 and *RPL29* using BIHIND-qPCR, and determined to be 85% for *AK2*, 81% for *PSMB2*, 95% for *ERH,* and 38% for *RPL29* (Figure 4E), respectively. Given TRUB1 has been shown as the Ψ writer for *ERH* Ψ649^14^, we proceeded to validate this result. Similarly, we conducted transient knockdown of TRUB1 in Hela cells using siRNA (Figure 4F), and observed a significant reduction in the Ψ fraction of *ERH* Ψ649 from 95% to 67% following TRUB1 depletion, as confirmed by BHIND-qPCR (Figure 4G). This confirms TRUB1’s role as the Ψ writer for *ERH* Ψ649, again showcasing the utility of BIHIND-qPCR in elucidating Ψ-related regulation even within low abundant mRNA transcripts.

**Figure 5.**
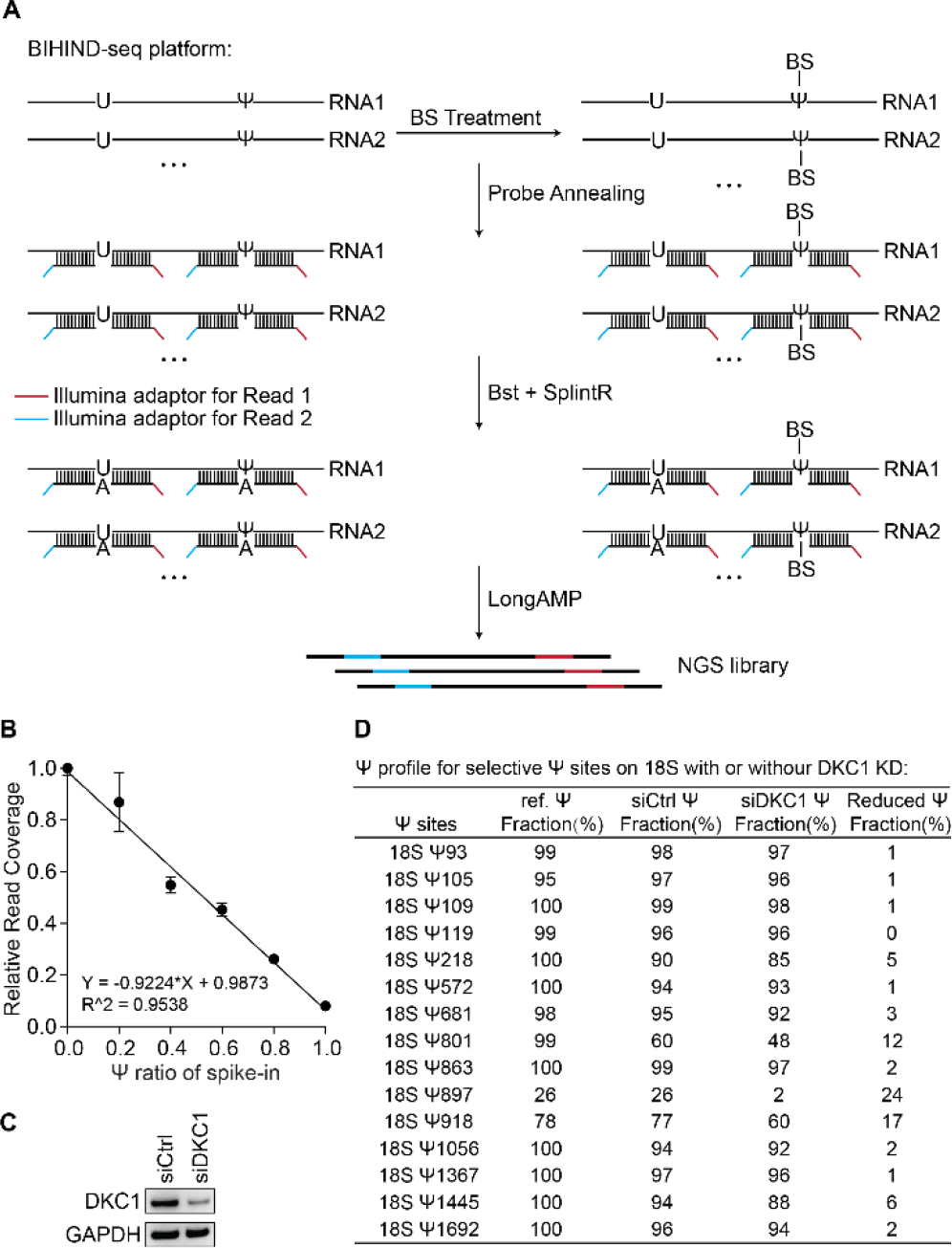
BIHIND-seq workflow and its application in Ψ quantification on rRNA. A) Illustrations of BIHIND-seq workflow. “Up-probes” and “Down-probes” with Illumina adaptors for putative Ψ sites were pooled together. After standard BIHIND protocol, the ligated product was amplified by LongAMP polymerase. B) The linear relationship between relative read coverage for spike-in and its Ψ fractions. C) Western blot showing reduced DKC1 expression level with siDKC1 treatment. D) BIHIND-seq results for Ψ fractions of selective Ψ sites on Hela 18S with or without DKC1 knockdown. Error bars, mean±s.d. for 2 biological replicates.

To further explore the potential of BIHIND, we tested when combining BIHIND with next-generation sequencing (NGS), or BIHIND-seq, for high-throughput detection of Ψ. We redesigned the “up-probe” and “down-probe” and added Illumina adaptor sequences for Read 1 and Read 2 to 5’-end of “up-probe” and 3’-end of “down-probe”, respectively. These adaptors facilitate PCR amplification of the ligated product, ultimately producing NGS libraries without the need for reverse transcription (Figure 5A). Optimization of PCR enzymes revealed that LongAMP produced the least background signal (Figure S4). To check feasibility of this principle, we spiked 25 ng of probes with various Ψ fractions into the 1.5 μg of total RNA, and calculated read coverage for each probe after library preparation and sequencing. As expected, the results demonstrated a negative linear relationship between read coverage and Ψ fractions (Figure 5B).

BIHIND-seq also shows great promises for functional studies in biological samples. Dyskeratosis Congenita 1 (DKC1), known to mediate the pseudouridylation of rRNA, is crucial for translational fidelity and has emerged as a potential prognostic biomarker^7,20–21^. To investigate the impact of DKC1 depletion on Ψ levels, we performed transient knockdown of *DKC1* in Hela cells (Figure 5C), and applied BIHIND-seq to monitor changes in the fraction of 15 selected Ψ sites (Figure 5D). The Ψ levels at these sites in wild-type Hela cells corresponded well with previously reported data on 18S Ψ sites^14^. After *DKC1* knockdown, fractions of most Ψ sites decreased, with 18S rRNA Ψ801 showing 20% decrease in the Ψ level, which was confirmed by a quantitative mass spectrometry approach^20^. Additionally, Ψ level change at 18S rRNA Ψ897 can be cross-validated by BIHIND-qPCR (Figure S5). These findings demonstrate that BIHIND-seq is a very useful method for high-throughput Ψ quantification for biological functional studies.

## Discussion

We report a locus-specific Ψ detection and quantification method based on bisulfite incorporation hindered ligation. BIHIND-qPCR can n be applied to detect Ψ sites using low input RNA down to 0.1 ng. its quantitative nature allowed us to study Ψ metabolism on mRNA and non-coding RNA with depletion of pseudouridine synthases or other perturbations. Very recently, Fang *et al* reported pseU-TRACE to achieve locus-specific pseudouridine detection^22^, based on the chemistry principle of RT-deletion-based BID-seq^14^. It is known that 100% Ψ in motif “UGΨAG” causes less than 70% deletion ratio during the RT process^14^. This incomplete deletion signature may underestimate Ψ ratio and requires a standard calibration curve. BIHIND-qPCR, on the other hand, employs an internal negative control for normalization. Both methods could be used for more accurate Ψ quantification.

## Conclusion

In conclusion, we report a straightforward method termed BIHIND for incorporating bisulfite into Ψ to discriminate between Ψ and U. This method, when combined with quantitative PCR (BIHIND-qPCR) or next-generation sequencing (BIHIND-seq), offers a convenient and robust method to identify Ψ sites and determine Ψ fractions across various RNA types (rRNA, lncRNA, and mRNA). By eliminating the need for CMC labeling of Ψ and simplifying purification, our approach facilitates Ψ detection even in RNA samples with limited input. We showed we can employ BIHIND to assign specific Ψ synthases responsible for individual Ψ installation, highlighting BIHIND’s potential as a robust tool for detecting Ψ in low-input RNA samples. This approach may also be used for biomarker detection in the future.

## Supplementary Material

SI

## Data Availability

The BIHIND-seq data have been deposited to NCBI GEO, accession number GSE269170.
