# Supplementary material for: Pseudouridine Detection and Quantification using Bisulfite Incorporation Hindered Ligation
Source: ACS Chem Biol. Author manuscript; Available in PMC 2025 Apr 30. (PMC12043245; doi:10.1021/acschembio.4c00387)
Supplement: SI [file NIHMS2072640-supplement-SI.pdf]

Supporting information

Pseudouridine Detection and Quantification using Bisulfite Incorporation Hindered Ligation

Yutao Zhao, Xinyuan Ma, Chang Ye, Wenlong Li, Kinga Pajdzik, Qing Dai, Hui-Lung Sun, and Chuan He*

Contents

Experimental Procedure 3
RefSeq ID used for BIHIND 3
MALDI-TOF MS 3
Cell Culture and RNA extraction 3
Western Blotting 3
Transient Knockdown 4
BIHIND-qPCR protocol 4
BIHIND-PAGE analysis on 41-mer RNA oligo 5
BIHIND-seq protocol 5
BIHIND-seq data analysis 6
Customed shell script “mapping.sh” 6
Supporting figures and tables 7
Figure S1a. 7
Figure S1b. 8
Figure S1c. 9
Figure S2. 9
Figure S3. 9
Figure S4. 10
Figure S5. 10
Table S1 10
Table S2 11
Table S3 12
Reference 13

Experimental Procedure

RefSeq ID used for BIHIND

RefSeq ID	Gene Symbol
RNA18SN5	NR_003286.4
RN7SK	NR_001445.2
MALAT1	NR_002819.4
AK2	NM_001625.4
ERH	NM_004450.3
PSMB2	NM_002794.5
RPL29	NM_000992.3

MALDI-TOF MS

We followed the reported protocol[1]. To 9 μL of BS reagent (prepared by mixing 270 mg Na_2SO_3 and 34 mg $NaHSO_3$ in 850 μL RNase-free water), 1 μL of synthetic RNA oligo AGXGA (X = C, U or Ψ, 100 ng/μL) was added, followed by mixing well via pipetting. The reaction mixture was incubated in PCR instrument for various temperatures and time (70 ^{o}C for 3 h; 80 ^{o}C for 2 h; 90 ^{o}C for 1 h). After cooling to room temperature, 10 μL Tris-HCl buffer (1.5 M, pH 8.8) was added and mixed well by pipetting. The mixture was incubated at 37 °C for 1 h for desulfonation. Then, 2 μL of the mixture was added to 40 μL resin (Bio-Rad) and allowed to stand at room temperature for 30 min. Then 1.8 μL supernatant was mixed with matrix 2′,4′,6′-trihydroxyacetophenone monohydrate and loaded onto a MALDI plate. The MALDI-TOF MS recorded the signals using negative reflector mode.

Cell Culture and RNA extraction

Hela, HEK293T and HepG2 cells were cultured in DMEM medium (Gibco, 11995073) containing 10% FBS (Gibco, 26140079) and 1% penicillin-streptomycin (Gibco, 15140122) at 37°C with 5% CO_2. Total RNA was extracted by TRIzol reagent (ThermoFisher Scientific, 15596026) according to the manufacturer's instructions. PolyA-RNA was isolated by two rounds of ployA enrichment with Dynabeads Oligo$(dT)_{25}$ (ThermoFisher Scientific, 61006) from total RNA.

Western Blotting

The protein level of PUS7, TRUB1 and DKC1 were measured by western blotting. In brief, siCtrl and siPUS7/siTRUB1/siDKC1 cell were harvested and mixed with RIPA buffer (ThermoFisher Scientific, 89901) for half an hour. Centrifuged to clear the lysate at 12000xg for 10 min. The clear lysate was added with a final conc of 1X NuPAGE™ LDS Sample Buffer (ThermoFisher Scientific, NP0007). Then incubated at 90 ^{o}C for 5 min. The samples were subjected to PAGE electrophoresis (ThermoFisher Scientific, NP0326BOX) and then transferred from gel to PVDF membrane. Antibody staining was carried out with anti-PUS7 antibody (Bethyl Laboratories, A305-146A), anti-TRUB1 antibody (Proteintech, 12520-1-AP) or anti-DKC1 antibody (Cell Signaling, 52234). Finally, membrane was imaged in iBright FL1500 Imaging System (ThermoFisher Scientific).

Transient Knockdown

Transient knockdown of PUS7/TRUB1/DKC1 was performed by siRNAs. In brief, Hela cell (60% confluent) was transfected with siCtrl (Qiagen, 1027310) and siPUS7 (Qiagen, SI04148361) /siTRUB1 (Millipore, SASI_HSO2_0036-4197) /siDKC1 (Qiagen, SI03080616) using Lipofectamine™ RNAiMAX Transfection Reagent (ThermoFisher Scientific, 13778075). Cells were harvested 48 h post-transfection.

BIHIND-qPCR protocol

<u>BS Treatment</u>: 5~10 μL Total RNA, polyA-RNA, or synthesized RNA oligos were mixed with 50 μL BS reagent (prepared by mixing 270 mg Na_2SO_3 and 34 mg $NaHSO_3$ in 850 μL RNase-free water). Incubate at 70 °C for 3 hours. Then added 95~90 μL water to dilute the sample and used EZ RNA Methylation Kit (Zymo, R5001) for purification. Briefly, added 250 μL RNA Binding buffer and 400 μL EtOH. Mixed well and kept it on ice for 30 min to achieve the best recovery. Then the mixture was transferred to a spin IC column and centrifuged at 12000xg for 1 min. The column was washed with 50 μL RNA wash buffer once. Added 40 μL RNA desulphonation buffer and incubated at room temperature for 1 h. Centrifuged the column at 12000xg for 1 min. The column was washed with 50 μL RNA wash buffer once. BS-treated RNA was eluted with 10 μL water.

<u>BIHIND-qPCR for 41-mer RNA oligo</u>: The same amount of 50 ng oligo with various Ψ fractions were mixed with two probes: 0.6 pmol "up probe" for Ψ and 0.6 pmol "down probe" for Ψ. The RNA and probes with total volume of 10 μL were annealed in 1X annealing buffer (10 mM Tris-HCl, pH 7.5, 50 mM NaCl, 1 mM EDTA) by incubating mixture at a temperature gradient: 90 °C for 1 min, 80 °C for 1 min, 70 °C for 1 min, 60 °C for 1 min, 50 °C for 1 min, 40 °C for 5 min and hold on 35 °C. Subsequently, a 10 μL of mixture containing 0.8 U *Bst* 2.0 DNA polymerase (NEB, M0537S), 0.1 nmol dATP in 2X CutSmart buffer was added to the former mixture. The reaction mixture was incubated at 45 °C for 30 min. Then another 10 μL of mixture containing 15% PEG8000, 6 mM ATP, 2.5 U SplintR Ligase (NEB, M0375S) and 1X CutSmart buffer was added and mixed well. The whole mixture was incubated at 25 °C for 20 min. 70 μL water was added to dilute the reaction mixture. Afterwards, quantitative PCR was performed on Roche LightCycler 96 system. 20 μL qPCR reaction was composed of 1X FastStart Essential DNA Green Master (Roche, 06402712001), 500 nM qPCR Ψ-detection F-primer, 500 nM qPCR Ψ-detection R-primer, 5 μL diluted reaction mixture. qPCR was run at the following condition: 95 °C, 5 min; (95 °C, 20 s; 60 °C, 20 s; 72 °C, 20 s)×40 cycles. C_t values were obtained from LightCycler 96 software.

<u>BIHIND-qPCR for cellular RNA</u>: BS-treated/Untreated total RNA or polyA-RNA were mixed with four probes: 60 nM "up probe" for U, 60 nM "up probe" for Ψ, 60 nM "down probe" for U and 60 nM "down probe" for Ψ. The RNA and probes with total volume of 10 μL were annealed in 1X annealing buffer (10 mM Tris-HCl, pH 7.5, 50 mM NaCl, 1 mM EDTA) by incubating mixture at a temperature gradient: 90 °C for 1 min, 80 °C for 1 min, 70 °C for 1min, 60 °C for 1 min, 50 °C for 1 min, 40 °C for 5 min and hold on 35 °C. Subsequently, a 10 μL of mixture containing 0.8 U *Bst* 2.0 DNA polymerase (NEB, M0537S), 0.1 nmol dATP in 2X CutSmart buffer was added to the former mixture. The reaction mixture was incubated at 45 °C for 20 min. Then another 10 μL of mixture containing 15% PEG8000, 6 mM ATP, 2.5 U SplintR Ligase (NEB,

M0375S) and 1X CutSmart buffer was added and mixed well. The whole mixture was incubated at 25 °C for 20 min. 70 μL water was added to dilute the reaction mixture. Afterwards, quantitative PCR was performed on Roche LightCycler 96 system. For BS-treated/Untreated RNA, 20 μL qPCR reaction was composed of 1X FastStart Essential DNA Green Master (Roche, 06402712001), 500 nM qPCR Ψ-detection F-primer (or qPCR U-detection F-primer), 500 nM qPCR Ψ-detection R-primer (or qPCR U-detection R-primer), 5 μL diluted reaction mixture. qPCR was run at the following condition: 95 °C, 5 min; (95 °C, 20 s; 60 °C, 20 s; 72 °C, 20 s)×40 cycles. C_t values were obtained from LightCycler 96 software.

BIHIND-PAGE analysis on 41-mer RNA oligo

BS Treatment: 2000 ng 41-mer RNA oligos with various Ψ fractions from 0% to 100% were mixed with 50 μL BS reagent (prepared by mixing 270 mg Na_2SO_3 and 34 mg $NaHSO_3$ in 850 μL RNase-free water). Incubate at 70 °C for 3 hours. Then added 85 μL water to dilute the sample and used EZ RNA Methylation Kit (Zymo, R5001) for purification. Briefly, added 250 μL RNA Binding buffer and 400 μL EtOH. Mixed well and kept it on ice for 30 min to get the best recovery. Then transferred the mixture to a spin IC column and centrifuged at 12000xg for 1 min. The column was washed with 50 μL RNA wash buffer once. Added 40 μL RNA desulphonation buffer and incubated at room temperature for 1 h. Centrifuged the column at 12000xg for 1 min. The column was washed with 50 μL RNA wash buffer once. BS-treated RNA was Eluted with 10 μL water. Measured the RNA concentration in nanodrop.

BIHIND-PAGE electrophoresis for 41-mer RNA oligo: 100 ng BS-treated oligo with various Ψ fractions were mixed with two probes: 1 μM FAM-labeled "up probe" and 1 μM "down probe". The RNA and probes with total volume of 10 μL were annealed in 1X annealing buffer (10 mM Tris-HCl, pH 7.5, 50 mM NaCl, 1 mM EDTA) by incubating mixture at a temperature gradient: 90 °C for 1 min, 80 °C for 1 min, 70 °C for 1 min, 60 °C for 1 min, 50 °C for 1 min, 40 °C for 5 min and hold on 35 °C. Subsequently, a 10 μL of mixture containing 0.8 U *Bst* 2.0 DNA polymerase (NEB, M0537S), 0.1 nmol dATP in 2X CutSmart buffer was added to the former mixture. The reaction mixture was incubated at 45 °C for 30 min. Then another 10 μL of mixture containing 15% PEG8000, 6 mM ATP, 2.5 U SplintR Ligase (NEB, M0375S) and 1X CutSmart buffer was added and mixed well. The whole mixture was incubated at 25 °C for 20 min. 30 μL 2X TBE-Urea Sample Buffer (ThermoFisher Scientific, LC6876) was mixed in the reaction mixture and then incubated at 90 °C for 5 min. 15 μL reaction mixture was loaded on a TBE-Urea Gel, 15% (ThermoFisher Scientific, EC68852BOX) and ran at 200 V for 0.5 h. The whole gel was imaged on BioRad ChemiDoc system.

BIHIND-seq protocol

1.5 μg BS-treated/Untreated total RNA were mixed with up-probes and down-probes (30 fmol each). The RNA and probes in total volume of 10 μL were annealed in 1X annealing buffer (10 mM Tris-HCl, pH 7.5, 50 mM NaCl, 1 mM EDTA) by incubating mixture at a temperature gradient: 90 °C for 1 min, 80 °C for 1 min, 70 °C for 1min, 60 °C for 1 min, 50 °C for 1 min, 40 °C for 5 min and hold on 35 °C. Subsequently, a 10 μL of mixture containing 0.8 U *Bst* 2.0 DNA polymerase (NEB, M0537S), 0.2 nmol dATP in 2X CutSmart buffer was added to the former mixture. The reaction mixture was incubated at 45 °C for 30 min. Then another 10 μL of mixture

containing 15% PEG8000, 1.5 nmol ATP, 2.5 U SplintR Ligase (NEB, M0375S) in 1X CutSmart buffer was added and mixed well. The whole mixture was incubated at 25 °C for 20 min. 20 μL water was added to dilute the reaction mixture. Ligated product was purified by Zymo RNA Clean & Concentrator-5 and eluted with 15 μL water. The eluted product was added with 7.5 μL water, 2.5 μL Unique Dual Index (NEB, E6442S) and 25 μL LongAmp Taq 2X Master Mix (2X) (NEB, M0287S). PCR was run at the following condition: 94 °C, 30 s; (94 °C, 15 s; 62 °C, 30 s; 70 °C, 15 s)×**C** cycles (**C**=12 for Untreated samples, **C**=14 for BS-treated samples); 70 °C, 5 min; 4 °C, hold-on. PCR product was purified by running 2.5% agarose TBE gel and recovered by Qiagen MinElute Gel Extraction Kit. The libraries were pooled in equal molarities and sequenced on one lane of an Illumina NextSeq 2000 platform. Around 0.5 M reads were acquired for each library.

BIHIND-seq data analysis

Only R1 reads of paired-end reads were processed for 18S Ψ site detection. The R1 reads were processed by following customed shell script called "mapping.sh". Briefly, 3' end of R1 was trimmed by cutadapt (version 4.8) μL[2], with following parameters "-j 15 -m 40 --max-n=0 -u 6 -e 0.15 -q 20 --discard-untrimmed --nextseq-trim=20 -a AGATCGGAAGAGCACACGTCTG". The trimmed reads were mapped to Ψ/U loci sequences by STAR mapping software (version 2.7.9a)[3] with setting "--outFilterMatchNmin 39" to minimize multiple mapping ratio. Software samtools (1.16.1-39-g1250b01)[4] was used to calculate read number mapped to each Ψ/U locus. Ψ ratio was calculated as $1 - \frac{(1-\text{ReadNumber}(\Psi\text{ locus for BS}-\text{treated library}))/(1-\text{ReadNumber}(\text{U locus for BS}-\text{library}))}{(1-\text{ReadNumber}(\Psi\text{ locus for untreated library}))/(1-\text{ReadNumber}(\text{U locus for untreated library}))}$.

Customed shell script "mapping.sh"

```
> cat mapping.sh

R1=$1

for i in ./mapping ./mapping/STAR_log ./log

do

	if [ ! -d $i ];then

		mkdir $i

	fi

done

cutadapt -j 15 -m 40 --max-n=0 -u 6 -e 0.15 -q 20 --discard-untrimmed --nextseq-trim=20 \

	-a AGATCGGAAGAGCACACGTCTG -o ${R1/.fastq.gz/.trimmed.fastq.gz}
$R1 > ./log/${R1/.R1.fastq.gz/}.log

STAR --runThreadN 10 --genomeDir BIHIND_rRNA/ --readFilesIn ${R1/.fastq.gz/.trimmed.fastq.gz} \

	--readFilesCommand zcat --outFilterMatchNmin 39 --alignEndsType Local \

	--outReadsUnmapped None --outSAMtype BAM Unsorted --
outFileNamePrefix ./mapping/${R1/.R1.fastq.gz/}_

cat ./mapping/${R1/.R1.fastq.gz/}_Log.final.out >> ./log/${R1/.R1.fastq.gz/}.log

mv ./mapping/${R1/.R1.fastq.gz/}_Log.final.out ./mapping/${R1/.R1.fastq.gz/}_Log.out \
```

```
./mapping/${R1/.R1.fastq.gz/}_Log.progress.out ./mapping/${R1/.R1.fastq.gz/}_SJ.out.tab ./mapping/STAR_log

rm ${R1/.fastq.gz/.trimmed.fastq.gz}

samtools sort -@ 10 -o ./mapping/${R1/.R1.fastq.gz/}.sorted.bam ./mapping/${R1/.R1.fastq.gz/}_Aligned.out.bam

rm ./mapping/${R1/.R1.fastq.gz/}_Aligned.out.bam

echo ${R1/.R1.fastq.gz/} >> total_num.txt

samtools view ./mapping/${R1/.R1.fastq.gz/}.sorted.bam | wc -l >> total_num.txt

samtools view ./mapping/${R1/.R1.fastq.gz/}.sorted.bam | awk '{a[$3]++}END {for (i in a){print i,a[i]}}' | sort -k1r > ./mapping/${R1/.R1.fastq.gz/}.read_num.txt

> bash mapping.sh xxx.R1.fastq.gz     ###(mapping R1 reads to fasta file)
```

Supporting figures and tables

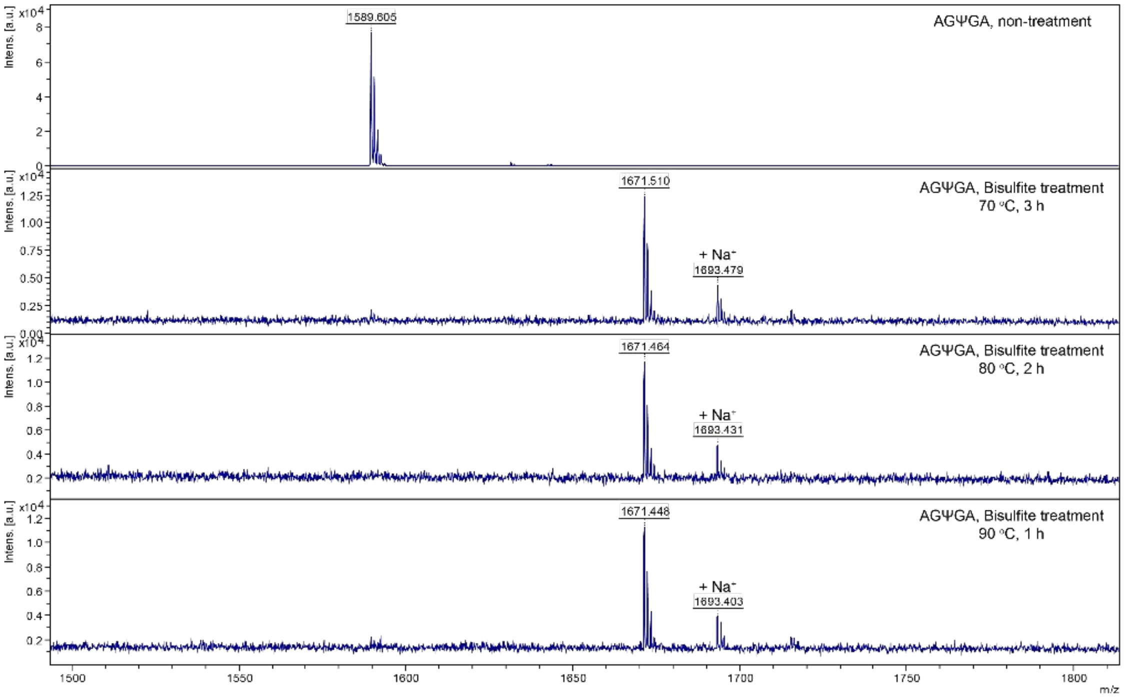

Figure S1a. MALDI-TOF MS results for BS-treated “AGΨGA” probe. “AGΨGA” probe was treated with BS reagent in different reaction conditions: 70 ^{o}C for 3 h; 80 ^{o}C for 2 h; 90 ^{o}C for 1 h.

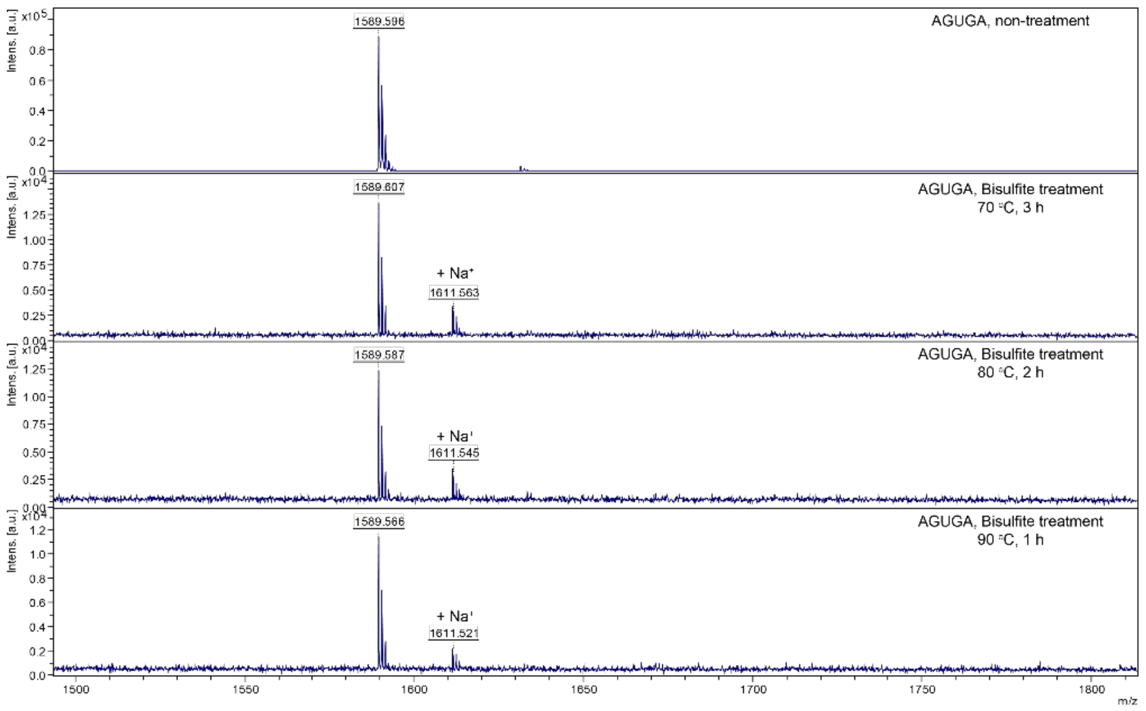

Figure S1b. MALDI-TOF MS results for BS-treated "AGUGA" probe. "AGUGA" probe was treated with BS reagent in different reaction conditions: 70 ^{o}C for 3 h; 80 ^{o}C for 2 h; 90 ^{o}C for 1 h.

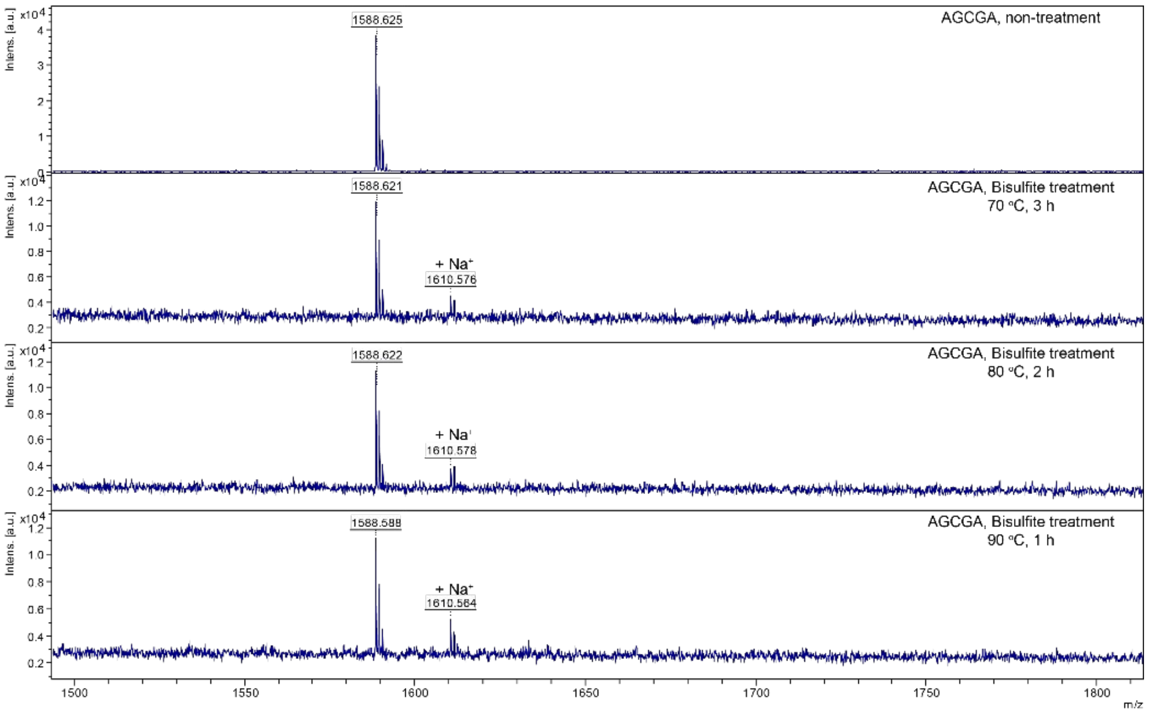

Figure S1c. MALDI-TOF MS results for BS-treated "AGCGA" probe. "AGCGA" probe was treated with BS reagent in different reaction conditions: 70 ^{o}C for 3 h; 80 ^{o}C for 2 h; 90 ^{o}C for 1 h.

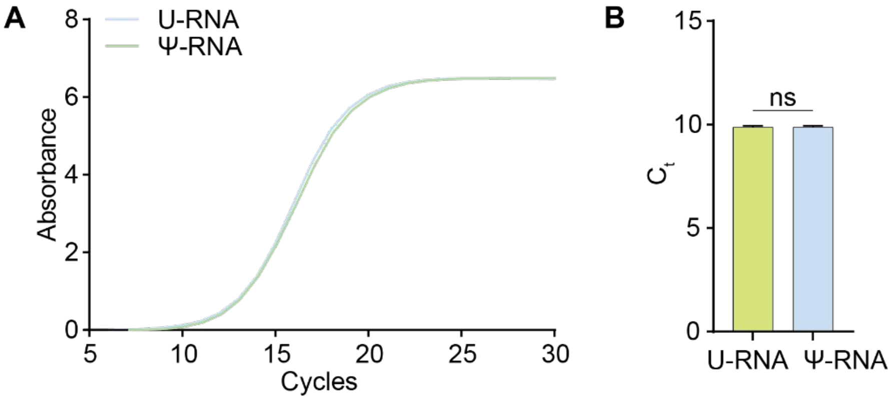

Figure S2. qPCR curve showing that SELECT method is not able to distinguish U/Ψ.

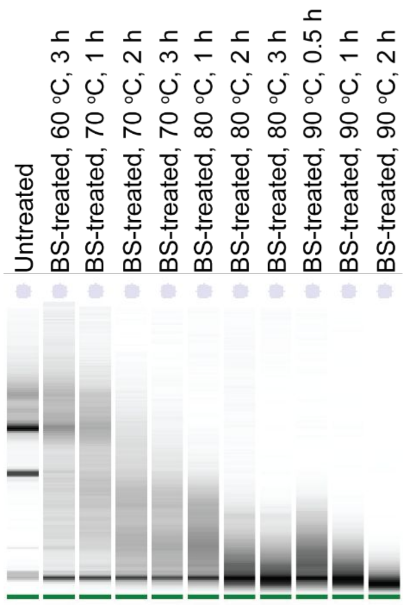

Figure S3. Evaluation of RNA degradation caused by BS treatment at different reaction temperature and different reaction time.

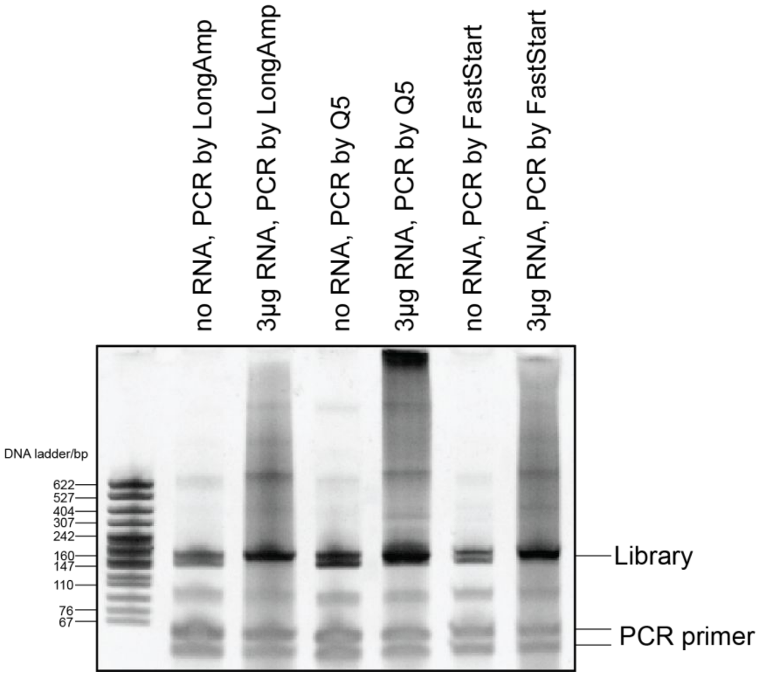

Figure S4. Optimization of PCR enzymes for BIHIND-seq.

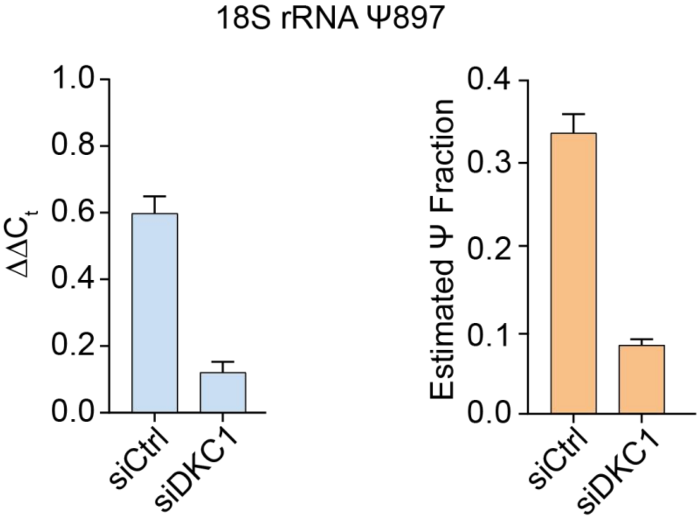

Figure S5. BIHIND-qPCR used for Ψ quantification of 18S rRNA Ψ897 with or without DKC1 knockdown. Error bars for 2 biological replicates x 3 technical replicates.

Table S1. Materials needed for BIHIND-qPCR of 41-mer RNA oligo.

	name	Sequence(5'->3')
41-mer RNA probe	41-mer Ψ-RNA probe	rUrUrGrGrArArArArGrGrArArCrUrCrUrUrGrGr**Ψ**rUrCrGrArUrArCrCrUrGrGrArGrCrArGrArGrGrArG
	41-mer U-RNA probe	rUrUrGrGrArArArArGrGrArArCrUrCrUrUrGrGr**U**rUrCrGrArUrArCrCrUrGrGrArGrCrArGrArGrGrArG
	up-probe-FAM	/56-FAM/CTCCTCTGCTCCAGGTATCGA
	down-probe	/5phos/CCAAGAGTTCCTTTTGGAA

	up-probe-qPCR	tagccagtaccgtagtgcgtgCTCCTCTGCTCCAGGTATCGA
	down-probe-qPCR	/5phos/CCAAGAGTTCCTTTTGGAAcagaggctgagtcgctgcat
	BIHIND-qPCR-F primer	ATGCAGCGACTCAGCCTCTG
	BIHIND-qPCR-R primer	TAGCCAGTACCGTAGTGCGTG

Table S2. Materials needed for BIHIND-qPCR of biological samples.

Ψ sites	name	Sequence(5'->3')
18S Ψ93	18S-93-down-probe	/5phos/TTCGCAGTTTCACTGTACCGcagaggctgagtcgctgcat
	18S-93-up-probe	tagccagtaccgtagtgcgtgCATAACTGATTTAATGAGCC
	N-18S-down-probe	/5phos/TTCCCCGTCACCCGTGGTCAtcgtatgccgtcttctgcttg
	N-18S-up-probe	aatgatacggcgaccaccgaCTCTCCGGAATCGAACCCTG
18S Ψ863	18S-863-down-probe	/5phos/TTCCTAGCTGCGGTATCCAGGcagaggctgagtcgctgcat
	18S-863-up-probe	tagccagtaccgtagtgcgtgGAACCGCGGTCCTATTCCATT
	N-18S-down-probe	/5phos/TTCCCCGTCACCCGTGGTCAtcgtatgccgtcttctgcttg
	N-18S-up-probe	aatgatacggcgaccaccgaCTCTCCGGAATCGAACCCTG
18S Ψ1056	18S-1056-down-probe	/5phos/TCGTCTTCGAACCTCCGACTcagaggctgagtcgctgcat
	18S-1056-up-probe	tagccagtaccgtagtgcgtgCGGAACTACGACGGTATCTG
	N-18S-down-probe	/5phos/TTCCCCGTCACCCGTGGTCAtcgtatgccgtcttctgcttg
	N-18S-up-probe	aatgatacggcgaccaccgaCTCTCCGGAATCGAACCCTG
18S Ψ897	18S-897-down-probe	/5phos/ACCAACAAAATAGAACCGCGcagaggctgagtcgctgcat
	18S-897-up-probe	tagccagtaccgtagtgcgtgATCATGGCCTCAGTTCCGAA
	N-18S-down-probe	/5phos/TTCCCCGTCACCCGTGGTCAtcgtatgccgtcttctgcttg
	N-18S-up-probe	aatgatacggcgaccaccgaCTCTCCGGAATCGAACCCTG
RN7SK Ψ250	7SK-250-down-probe	/5phos/CAAATGGACCTTGAGAGCTTcagaggctgagtcgctgcat
	7SK-250-up-probe	tagccagtaccgtagtgcgtgGACTACCCTACGTTCTCCT
	N-7SK-down-probe	/5phos/CCGAAGACCGGTCCTCCTCTtcgtatgccgtcttctgcttg
	N-7SK-up-probe	aatgatacggcgaccaccgaCAGCTACTCGTATACCCTTG
MALAT1 Ψ5614	MALAT1-5614-down-probe	/5phos/CAATTTTAAAAAGGCTCGATcagaggctgagtcgctgcat
	MALAT1-5614-up-probe	tagccagtaccgtagtgcgtgAGCCCACAGGAACAAGTCCT
	N-MALAT1-down-probe	/5phos/ACTCTTCTGATAACGAAGAGtcgtatgccgtcttctgcttg
	N-MALAT1-up-probe	aatgatacggcgaccaccgaGCTCCCAGATGAAATGAAGC
AK2	AK2-down-probe	/5phos/ACTCCTCGTGGTAGGAACGGcagaggctgagtcgctgcat
	AK2-up-probe	tagccagtaccgtagtgcgtgTGGGCTCTTTTGGAGGGTTG
	N-AK2-down-probe	/5phos/ATTCAATCACAGAATCAAGCtcgtatgccgtcttctgcttg
	N-AK2-up-probe	aatgatacggcgaccaccgaGCAGAGAGTCTGGGATGCTG
ERH	ERH-down-probe	/5phos/ACCAACATTAAGTGACGAAGcagaggctgagtcgctgcat
	ERH-up-probe	tagccagtaccgtagtgcgtgGATATGAACAGTTGAAGGACTGG
	N-ERH-down-probe	/5phos/AATCAAACAACTGACTGATGTtcgtatgccgtcttctgcttg
	N-ERH-up-probe	aatgatacggcgaccaccgaGGTCTGCCAGATCATCGATG
PSMB2	PSMB2-down-probe	/5phos/ACACTGAAGGTTGGCAGATTCcagaggctgagtcgctgcat
	PSMB2-up-probe	tagccagtaccgtagtgcgtgGCCATTTTTGTCAATGATTCG
	N-PSMB2-down-probe	/5phos/AGCCAAGCATGGAGTAGAACGtcgtatgccgtcttctgcttg
	N-PSMB2-up-probe	aatgatacggcgaccaccgaGAAAGAGCTAGTTGGAAAGG
RPL29	RPL29-down-probe	/5phos/CGGGCACGAGCACGCTTCCCcagaggctgagtcgctgcat

	RPL29-up-probe	tagccagtaccgtagtgcgtgGCCTGAGCCCCTTGGCAAT
	N-RPL29-down-probe	/5phos/CAAATAGCACAGGAGGACCCtcgtatgccgtcttctgcttg
	N-RPL29-up-probe	aatgatacggcgaccaccgaCCTGCCTCAGGTTTATTTGT
qPCR primer	BIHIND-qPCR-F primer	ATGCAGCGACTCAGCCTCTG
	BIHIND-qPCR-R primer	TAGCCAGTACCGTAGTGCGTG
	N-BIHIND-qPCR-F primer	CAAGCAGAAGACGGCATACGA
	N-BIHIND-qPCR-R primer	AATGATACGGCGACCACCGA

Table S3. Materials needed for BIHIND-seq of biological samples.

41-mer Ψ-RNA probe (Ψ spike-in)	rUrUrGrGrArArArArGrGrArArCrUrCrUrUrGrGr**Ψ**rUrCrGrArUrArCrCrUrGrGrArGrCrArGrArGrGrArG
41-mer U-RNA probe (U spike-in)	rUrUrGrGrArArArArGrGrArArCrUrCrUrUrGrGr**U**rUrCrGrArUrArCrCrUrGrGrArGrCrArGrArGrGrArG
oligo-down-probe	/5Phos/CCAAGAGTTCCTTTTGGAAAGATCGGAAGAGCACACGTCTGAACTCCAGTCAC
oligo-up-probe-N	ACACTCTTTCCCTACACGACGCTCTTCCGATCTNNNNNNCTCCTCTGCTCCAGGTATCGA
N-18S-361-down-probe	/5Phos/TAGGGCAGACGTTCGAATGGAGATCGGAAGAGCACACGTCTGAACTCCAGTCAC
N-18S-361-up-probe	ACACTCTTTCCCTACACGACGCTCTTCCGATCTNNNNNNGCGACTACCATCGAAAGTTG
18S-93-down-probe	/5Phos/TTCGCAGTTTCACTGTACCGAGATCGGAAGAGCACACGTCTGAACTCCAGTCAC
18S-93-up-probe-N	ACACTCTTTCCCTACACGACGCTCTTCCGATCTNNNNNNCATAACTGATTTAATGAGCC
18S-105-down-probe	/5Phos/TTTAATGAGCCATTCGCAGTAGATCGGAAGAGCACACGTCTGAACTCCAGTCAC
18S-105-up-probe-N	ACACTCTTTCCCTACACGACGCTCTTCCGATCTNNNNNNGACCAAAGGAACCATAACTG
18S-109-down-probe	/5Phos/CTGATTTAATGAGCCATTCGAGATCGGAAGAGCACACGTCTGAACTCCAGTCAC
18S-109-up-probe-N	ACACTCTTTCCCTACACGACGCTCTTCCGATCTNNNNNNGAGCGACCAAAGGAACCATA
18S-119-down-probe	/5Phos/GGAACCATAACTGATTTAATAGATCGGAAGAGCACACGTCTGAACTCCAGTCAC
18S-119-up-probe-N	ACACTCTTTCCCTACACGACGCTCTTCCGATCTNNNNNNGAGAGGAGCGAGCGACCAA
18S-218-down-probe	/5Phos/TGCACGCATCCCCCCCGCGAAGATCGGAAGAGCACACGTCTGAACTCCAGTCAC
18S-218-up-probe-N	ACACTCTTTCCCTACACGACGCTCTTCCGATCTNNNNNNGGTTGGTTTTGATCTGATAA
18S-572-down-probe	/5Phos/AGTGGACTCATTCCAATTACAGATCGGAAGAGCACACGTCTGAACTCCAGTCAC
18S-572-up-probe-N	ACACTCTTTCCCTACACGACGCTCTTCCGATCTNNNNNNGATCCTCGTTAAAGGATTTA
18S-681-down-probe	/5Phos/CTACGAGCTTTTTAACTGCAAGATCGGAAGAGCACACGTCTGAACTCCAGTCAC
18S-681-up-probe-N	ACACTCTTTCCCTACACGACGCTCTTCCGATCTNNNNNNCCGCCCGCTCCCAAGATCCA
18S-801-down-probe	/5Phos/AACGCTTCGGGCCCCGCGGGAGATCGGAAGAGCACACGTCTGAACTCCAGTCAC
18S-801-up-probe-N	ACACTCTTTCCCTACACGACGCTCTTCCGATCTNNNNNNCACTCTAATTTTTTCAAAGT
18S-863-down-probe	/5Phos/TTCCTAGCTGCGGTATCCAGGAGATCGGAAGAGCACACGTCTGAACTCCAGTCAC
18S-863-up-probe-N	ACACTCTTTCCCTACACGACGCTCTTCCGATCTNNNNNNGAACCGCGGTCCTATTCCATT
18S-897-down-probe	/5Phos/ACCAACAAAATAGAACCGCGAGATCGGAAGAGCACACGTCTGAACTCCAGTCAC
18S-897-up-probe-N	ACACTCTTTCCCTACACGACGCTCTTCCGATCTNNNNNNATCATGGCCTCAGTTCCGAA
18S-918-down-probe	/5Phos/ATCATGGCCTCAGTTCCGAAAGATCGGAAGAGCACACGTCTGAACTCCAGTCAC
18S-918-up-probe-N	ACACTCTTTCCCTACACGACGCTCTTCCGATCTNNNNNNTGCCCCCGGCCGTCCCTCTT
18S-1056-down-probe	/5Phos/TCGTCTTCGAACCTCCGACTAGATCGGAAGAGCACACGTCTGAACTCCAGTCAC
18S-1056-up-probe-N	ACACTCTTTCCCTACACGACGCTCTTCCGATCTNNNNNNCGGAACTACGACGGTATCTG
18S-1367-down-probe	/5Phos/CCAGACAAATCGCTCCACCAAGATCGGAAGAGCACACGTCTGAACTCCAGTCAC
18S-1367-up-probe-N	ACACTCTTTCCCTACACGACGCTCTTCCGATCTNNNNNNCTCGTTCGTTATCGGAATTA
18S-1445-down-probe	/5Phos/AGAAGTTGGGGGACGCCGACAGATCGGAAGAGCACACGTCTGAACTCCAGTCAC
18S-1445-up-probe-N	ACACTCTTTCCCTACACGACGCTCTTCCGATCTNNNNNNTGAACGCCACTTGTCCCTCT

18S-1136-down-probe	/5Phos/GCTGCCCGGCGGGTCATGGGAGATCGGAAGAGCACACGTCTGAACTCCAGTCAC
18S-1692-up-probe-N	ACACTCTTTCCCTACACGACGCTCTTCCGATCTNNNNNNTAGCGACGGGCGGTGTGTAC

Reference

[1] Q. Dai, L. S. Zhang, H. L. Sun, K. Pajdzik, L. Yang, C. Ye, C. W. Ju, S. Liu, Y. Wang, Z. Zheng, L. Zhang, B. T. Harada, X. Dou, I. Irkliyenko, X. Feng, W. Zhang, T. Pan, C. He, *Nat Biotechnol* **2023**, *41*, 344-354.

[2] M. Martin, *EMBnet.journal* **2011**, *17*, 3.

[3] A. Dobin, C. A. Davis, F. Schlesinger, J. Drenkow, C. Zaleski, S. Jha, P. Batut, M. Chaisson, T. R. Gingeras, *Bioinformatics* **2013**, *29*, 15-21.

[4] H. Li, B. Handsaker, A. Wysoker, T. Fennell, J. Ruan, N. Homer, G. Marth, G. Abecasis, R. Durbin, S. Genome Project Data Processing, *Bioinformatics* **2009**, *25*, 2078-2079.
